# OptOrch: a modular optimization toolkit for forest tree seed orchard deployment

**DOI:** 10.1093/g3journal/jkag208

**Published:** 2026-08-01

**Authors:** Ye-Ji Kim, Václav Bittner, Kyu-Suk Kang, Christi Sagariya, Milan Lstibůrek

**Affiliations:** Faculty of Forestry and Wood Sciences, Czech University of Life Sciences Prague, Praha 6 165 21, Czech Republic; Faculty of Science, Humanities and Education, Technical University of Liberec, Liberec 1 461 17, Czech Republic; Department of Agriculture, Forestry and Bioresources, College of Agriculture and Life Sciences, Seoul National University, Seoul 08826, Republic of Korea; Faculty of Forestry and Wood Sciences, Czech University of Life Sciences Prague, Praha 6 165 21, Czech Republic; Faculty of Forestry and Wood Sciences, Czech University of Life Sciences Prague, Praha 6 165 21, Czech Republic

**Keywords:** forest tree breeding, seed orchard deployment, optimal contribution selection, genetic coancestry, AMPL, mathematical programing

## Abstract

OptOrch is a transparent and modular optimization toolkit for forest tree seed orchard (SO) deployment. It implements SO-specific optimal contribution models in AMPL (A Mathematical Programing Language), separating biological assumptions, input data, and numerical solvers. Unlike existing optimal-contribution or mate-allocation software, OptOrch exposes the objective function and constraints directly, allowing breeders to modify SO-specific requirements, including status-number thresholds, proportional contribution bounds, graft availability, pairwise coancestry restrictions, female and male gametic contributions, and external pollen flow. When the declared formulation is supported by the selected solver, solutions can be evaluated using solver-reported feasibility status, objective bounds, and optimality gaps. The algebraic framework also allows alternative formulations to be tested when biological constraints create mixed-integer, nonlinear, or nonconvex instances. We demonstrate OptOrch with simulation-based scenarios involving alternative status-number thresholds and pollen contamination. The framework quantifies gain–diversity trade-offs, evaluates external pollen effects, and compares feasible deployment strategies under realistic biological and operational constraints.

## Introduction

Seed orchards (SOs) are a central component of forest tree improvement programs because they enable the large-scale production of genetically improved forest reproductive material (Funda and El-Kassaby 2012). In conventional clonal SOs, selected genotypes (referred to as “clones”) are vegetatively propagated, usually by grafting, and deployed in proportions to deliver genetic gain while maintaining sufficient genetic diversity in the resulting seed crop. This balance is essential because the exclusive deployment of the highest-ranking genotypes can increase short-term gain but may also increase the risk of inbreeding in deployed material.

Unlike many animal-breeding allocation problems, where selected parents can be assigned to specific matings or numbers of offspring, clonal SO deployment usually optimizes the composition of the mating population itself. Breeders decide which clones to include and how many ramets (grafts) of each clone to establish, while subsequent seed crops are generally produced through open pollination, with mating assumed to occur approximately at random among reproductively active orchard parents. Consequently, the optimized contribution is an expected contribution to future seed crops rather than a directly assigned mating or offspring count, and the realized genetic composition may be modified by fertility variation, flowering synchrony, pollen contamination, supplemental pollination, mortality, and orchard management.

The problem of optimizing genetic contributions originated largely in animal breeding. Early approaches focused on maximizing genetic gain under predefined inbreeding restrictions (Meuwissen 1997), followed by numerical optimization methods capable of handling multiple constraints simultaneously (Kinghorn 1998; Fernández and Toro 1999; Pong-Wong and Woolliams 2007). In forestry, the theoretical basis for unequal clonal deployment was established by Lindgren and Matheson (1986) and Lindgren et al. (1989), who showed that optimal contributions scale linearly with breeding values when constrained by status number, a diversity measure based on group coancestry, assuming unrelated clones. In practice, however, SO deployment often involves related candidates, finite orchard size, biological limits on reproductive output, and operational restrictions on the minimum and maximum representation of individual clones. These features make simplified analytical solutions insufficient for routine decision support (Lindgren et al. 1993).

More flexible mathematical programing approaches have therefore been explored in forestry, including mathematical programing for optimizing SO crops (Funda et al. 2009) and semidefinite programing (SDP) for unequal clonal deployment (Ahlinder et al. 2014). These studies provide important foundations, but their practical scope differs from the purpose of OptOrch. The approach of Funda et al. (2009) was developed for optimizing the composition of selected seed-crop subsets using observed reproductive output, and is therefore closely tied to crop-specific inputs and selective harvesting decisions. The SDP approach of Ahlinder et al. (2014) addresses unequal clonal deployment and contribution bounds, but its implementation is organized around a specific SDP formulation and solver workflow. Simpler linear restrictions on parental contributions have also been proposed for particular forestry breeding contexts (Lstibůrek et al. 2015). Such formulations can be useful when the biological setting permits a simplified representation of relatedness, but they do not replace the need for a flexible framework in which group coancestry, contribution bounds, pairwise restrictions, sex-specific gametic contributions, and pollen-flow assumptions can be expressed explicitly. General-purpose software is also available for related tasks, including optimal contribution selection and mate allocation. For example, optiSel (Wellmann 2019) provides an R framework for optimum contribution selection and mate allocation, and AlphaMate (Gorjanc and Hickey 2018) optimizes selection, maintenance of diversity, and mate allocation in breeding programs. These tools are valuable for their intended applications, but they are not designed specifically around clonal SO deployment under open pollination, where breeders optimize orchard composition before future mating occurs and may need to revise the deployment model itself as biological or operational assumptions change. This motivates a more flexible SO-specific framework in which breeders can adapt the model at the level of algebraic formulation, while leaving the numerical solution to an appropriate optimization solver.

This need makes SO deployment a natural application for algebraic mathematical programing. Rather than embedding one fixed optimization algorithm in software, an algebraic modeling language allows the breeder to declare the objective function, decision variables, parameters, and constraints in a form close to mathematical notation. This distinction is important because practical SO deployment is rarely a single invariant problem. One breeding program may require only a status-number constraint and upper bounds on clonal contributions, whereas another may need additional restrictions on pairwise coancestry, graft availability, female and male gametic contributions, dioecious reproductive biology, pollen contamination, or minimum representation of previously established material. An algebraic formulation allows these requirements to be added, removed, or modified as explicit equations, while the numerical solver remains a separate component chosen according to the mathematical structure of the resulting optimization instance.

This separation between model declaration and numerical solution is central to OptOrch. When the declared formulation belongs to a solver-supported class, such as a continuous convex, mixed-integer linear, or mixed-integer quadratic formulation, the solver can report feasibility, objective bounds, and optimality gaps relative to a user-specified tolerance. Thus, the user can distinguish between a feasible deployment strategy, an infeasible set of biological or operational requirements, and a solution that is certified to be within a declared tolerance of the best bound available to the solver. At the same time, not all biologically motivated extensions are equally tractable. Logical pairwise exclusions, integer ramet allocation, or nonlinear descriptions of fertility and contamination may produce mixed-integer or nonconvex formulations. In such cases, the value of an algebraic framework is that these assumptions remain visible and testable: breeders can compare alternative formulations, change bounds or diversity thresholds, evaluate solver behavior, and diagnose which constraints drive infeasibility or loss of gain.

Here we present OptOrch, a publicly available modeling toolkit for SO deployment optimization. OptOrch is implemented in AMPL (A Mathematical Programing Language; Fourer and Gay 2002; Fourer et al. 2003), which provides the algebraic modeling layer, while the numerical solver remains interchangeable. The framework uses estimated breeding values and pedigree- or marker-derived relationship matrices as primary inputs and determines optimal clonal contributions subject to explicit constraints on genetic diversity and operational feasibility. The core model maximizes expected genetic gain under a status-number constraint and contribution bounds. Additional AMPL model variants illustrate how breeders can formulate SO-specific extensions, including pairwise coancestry constraints, separate female and male contributions, and external pollen flow. Companion R (R Core Team 2025) scripts are used to generate inputs, automate scenario analyses, and process outputs, but the optimization model itself is defined in AMPL.

In the present study, we demonstrate OptOrch using simulation-based SO deployment scenarios. We first evaluate how alternative status-number thresholds affect optimal clonal contributions and expected genetic response. We then illustrate how external pollen contamination can be incorporated into the deployment model and used to assess its effect on expected gain. These examples are intended to demonstrate OptOrch as a decision-support framework for comparing feasible deployment strategies, exploring gain–diversity trade-offs, and adapting the optimization formulation to program-specific biological and operational requirements.

## Methods

### OptOrch implementation and optimization framework

As noted previously, a key advantage of using AMPL is the separation of model specification and data into two distinct files. This design allows breeders to readily apply existing optimization models to their own datasets and to modify the models as needed, without having to worry about data integration. To improve clarity and conciseness, we present the optimization model using matrix notation. The AMPL code is provided here in its basic mathematical form, emphasizing intuition rather than requiring extensive technical expertise. For readers interested in exploring the details of AMPL coding more deeply, the book by Fourer et al. (2003) is an excellent resource. AMPL is used only for algebraic model declaration and problem instantiation. The numerical solution is obtained by the selected solver, and the interpretation of optimality depends on the mathematical class of the instantiated model and the solver settings. For solver-supported formulations, the solver reports feasibility status, objective bounds, and an optimality gap relative to the specified tolerance. These diagnostics are therefore part of the OptOrch output and should be considered together with the optimized contribution vector.

OptOrch is organized into three complementary layers. First, the optimization model itself is defined in AMPL, where the objective function, variables, and constraints are written in algebraic form. This AMPL layer constitutes the core software contribution of the present study. Second, a numerical solver is required to solve the instantiated optimization problem. In this paper, we used Gurobi (Gurobi Optimization, LLC 2026) as the solver backend. Third, companion R scripts were used to generate simulated data, prepare scenario-specific inputs, launch repeated analyses, and summarize outputs.

The same underlying optimization problem can therefore be executed through two different computational pathways. In the primary workflow, AMPL reads the model and data files and passes the instantiated problem to Gurobi. In the alternative workflow, the same optimization problem is assembled directly in R using Gurobi-specific data structures and solver calls. The latter route is useful for benchmarking and solver-level experimentation, but it is inherently more verbose and less transparent because the user must explicitly construct objective vectors, variable types, bounds, linear constraints, and quadratic constraint objects in a solver-specific format. We emphasize this distinction because the main purpose of OptOrch is not to provide a solver-specific R implementation, but rather a transparent and modifiable AMPL-based optimization toolkit that can be maintained independently of a particular solver-specific application programing interface (API). In the public repository, the AMPL files document the general algebraic formulation and provide a minimal runnable example, whereas the R-based solver-specific scripts using Gurobi provide a complete reproducible workflow for the simulation scenarios and figures presented in this manuscript.

In this section, we formulate a general optimization model in the OptOrch_core.mod file, which comprises the declaration of parameters, sets, and variables, the construction of the objective function, and constraints (Box 1).

Box 1 **OptOrch_core.mod**

**Table:** 

#Parameters and sets	
param nc > 0;	1
set S := 1..nc by 1;	2
param ns > 0;	3
param b {i in S};	4
param l {i in S} >= 0, <= 1;	5
param u {i in S} >= 0, <= 1;	6
param c {i in S, j in S};	7
#Variables	
var p {i in S} >= 0, <= 1;	8
var r {i in S} binary;	9
#Objective function	
maximize gain: sum{i in S} b[i] * p[i];	10
#Constraints	
s.t. statusnumber: sum {i in S,j in S}	
c[i,j] * p[i] * p[j] <= 1/(2*ns);	11
s.t. totalcontribution: sum {i in S} p[i] = 1;	12
s.t. lowerbound {i in S}: p[i] >= r[i] * l[i];	13
s.t. upperbound {i in S}: p[i] <= r[i] * u[i];	14

#### Parameters and sets

A candidate population consists of $n_{c}$ individuals (line 1), numerically represented by the set *S* of integers from 1 to $n_{c}$ in ascending order (line 2). The parameter $n_{s}$ denotes the specified minimum Status Number ($n_{s}$, line 3), which is a diversity measure derived from group coancestry and can be interpreted as the effective number of unrelated, equally contributing parents that would produce the same level of group coancestry (Lindgren et al. 1996). Larger $n_{s}$ values impose stricter diversity requirements and generally require genetic contributions to be distributed across more candidates, whereas smaller $n_{s}$ values allow stronger concentration of contributions among high-ranking candidates. The vector *b* contains the breeding values of all selection candidates (line 4). Alternatively, an aggregate breeding value (multi-trait index) may be used as the selection criterion. The vectors *l* and *u* define the lower and upper bounds on proportional clonal contributions, respectively (lines 5–6), where each value represents the minimum and maximum allowable fraction of the total orchard contribution assigned to candidate *i*. The matrix *c* is the $n_{c}\times n_{c}$ genetic coancestry matrix for all selection candidates (line 7).

#### Variables

We assume clonal contributions p∈[0,1], i.e. the variable to be optimized (line 8). An auxiliary binary variable, represented as *r* (line 9), is incorporated within a constraint that will be discussed subsequently.

The binary variable $r_{i}$ acts as a selection indicator for candidate *i*. When $r_{i}=0$, the upper-bound constraint forces $p_{i}=0$, so the candidate is not selected. When $r_{i}=1$, the lower- and upper-bound constraints require $l_{i}\leq p_{i}\leq u_{i}$, so the selected candidate must contribute within the specified proportional bounds.

#### Objective function

The objective (line 10) is to maximize the product of breeding values and respective clonal contributions (genetic gain, ΔG), i.e.


(1) $$\text{maximize}\;\Delta G=\sum_{i=1}^{n_{c}}b_{i}p_{i}$$

#### Constraints

The quadratic constraint “statusnumber” (line 11) is set to limit the $n_{s}$ of the selected set to conform to the prescribed limit (input parameter $n_{s}$), i.e.


(2) $$\sum_{i=1}^{n_{c}}\sum_{\;j=1}^{n_{c}}c_{ij}p_{i}p_{\;j}\leq \frac{1}{2n_{s}}$$

If available, the genomic relationship matrix (G) may be utilized in place of *c* as noted by Woolliams et al. (2015). However, to ensure compatibility with Equation 2, the genomic matrix must be scaled by a factor of 0.5. The analyses presented in this manuscript use the group coancestry constraint based on status number in Equation 2. Alternative diversity constraints can be incorporated by modifying the AMPL model when appropriate for a given deployment problem, but they are not used in the present case-study analyses.

In the core model, the sum of all contributions is one (constraint “totalcontribution,” line 12), i.e.


(3) $$\sum_{i=1}^{n_{c}}p_{i}=1.$$

The selection-dependent contribution bounds are implemented using the auxiliary binary variable $r_{i}$:


(4) $$p_{i}\geq r_{i}l_{i},\;p_{i}\leq r_{i}u_{i},\;r_{i}\in \{0,1\},\;\forall \;i\in S.$$

When $r_{i}=0$, the lower- and upper-bound constraints force $p_{i}=0$, so candidate *i* is not selected. When $r_{i}=1$, the lower- and upper-bound constraints require $l_{i}\leq p_{i}\leq u_{i}$, so the selected candidate must contribute within the specified bounds. Equivalently, each contribution satisfies


(5) $$p_{i}=0\;\text{or}\;l_{i}\leq p_{i}\leq u_{i}.$$

The bounds $l_{i}$ and $u_{i}$ can be specified either as common values across individuals or as individual-specific values to reflect practical considerations, such as ramet availability or propagation capacity.

The model optimizes continuous clonal proportions. For practical deployment, these optimized proportions can subsequently be converted into integer counts based on the target SO size, while preserving the total number of vegetative copies (grafts) and minimizing deviations from the optimized genetic gain and diversity levels.

### Scope of optimization formulations and solver guarantees

OptOrch separates the biological formulation of the SO deployment problem from the numerical algorithm used to solve it. The AMPL model file specifies the sets, parameters, decision variables, objective function, and constraints, whereas the selected solver determines how the resulting optimization instance is solved. This distinction is important because different user-defined SO scenarios may lead to different mathematical problem classes. The core formulation, with continuous clonal contributions, contribution bounds, and a quadratic group coancestry constraint, can be treated as a continuous quadratic-constrained optimization problem when the relationship matrix is positive semidefinite and all other constraints remain linear. Adding selection-dependent lower bounds, exact integer ramet allocation, or logical pairwise restrictions may convert the problem into a mixed-integer formulation. Other extensions, such as nonlinear fertility or contamination functions, may lead to nonconvex formulations.

Accordingly, OptOrch does not assume that all possible user-defined extensions are equally tractable. Instead, the framework makes the formulation explicit and allows users to choose a solver appropriate for the resulting problem class. For solver-supported formulations, the solver returns diagnostic information such as feasibility status, objective bounds, and optimality gap relative to a declared tolerance. These diagnostics are reported with the solution and should be interpreted together with the biological assumptions and constraints used to define the optimization instance.

### Tailoring the model to seed orchard applications

In this section, we present a selection of potential modifications that can be implemented to optimize the model for addressing particular scenarios in SOs. While this compilation is by no means exhaustive, we encourage breeders to build upon these suggestions and tailor them to their unique needs.

#### Adjustments of the selection criteria

OptOrch treats $b_{i}$ as the user-supplied scalar selection criterion for candidate *i*. In the examples presented here, $b_{i}$ corresponds to the genomic estimated breeding value (GEBV). However, the same optimization framework can also be applied to other scalar deployment criteria defined before optimization, such as a multi-trait selection index or an empirically adjusted value that incorporates prediction uncertainty (Hickey et al. 2009; Resende et al. 2012) as well as additional biological or operational information.

Two distinct inbreeding-related issues should be separated. First, relatedness among selected orchard parents can increase the expected inbreeding of the resulting seed crop under open pollination (Funda and El-Kassaby 2012). This issue is addressed directly in OptOrch through the group coancestry with status number constraint and, when appropriate, through additional pairwise coancestry restrictions. Second, a candidate parent may itself be inbred. If such parental inbreeding reduces vigor, fertility, flowering, seed production, pollen production, or another component of reproductive output (Wu et al. 2004), then the expected contribution or deployment value of that candidate may differ from its breeding value for the target trait. When supported by empirical data or by the breeding objective, users may incorporate such information by modifying the input selection criterion $b_{i}$, by imposing candidate-specific contribution bounds, or by extending the model to include female- and male-specific reproductive constraints.

OptOrch does not prescribe a universal correction for prediction uncertainty or parental inbreeding effects. Such adjustments should be justified from the genetic evaluation model, empirical reproductive data, or program-specific deployment objective. No uncertainty- or parental-inbreeding-based adjustment of $b_{i}$ was applied in the analyses presented here.

#### Constraining pairwise coancestry

Although the group coancestry constraint in Equation 2 controls the overall diversity of the selected set, breeders are often additionally concerned about pairwise coancestry between individual clones. This is particularly relevant in SO deployment, because simultaneous selection of closely related clones may increase the risk of inbreeding in the resulting SO crop. To address this, OptOrch can impose an upper bound $c_{u}$ on acceptable pairwise coancestry among selected clones. Alternative AMPL formulations of this constraint are provided in the Supplementary Material.

#### Female and male contributions

To address various practical situations, it is beneficial to divide clonal contribution *p* into its corresponding female and male components. Consequently, we introduce two supplementary variables representing female f∈[0,1] and male m∈[0,1] contributions. The clonal contribution of parent *i* is then defined as


(6) $$p_{i}=(f_{i}+m_{i})/2\;\forall \;i\in S$$

Further implementation details and the complete model are provided in the Supplementary Material.

#### Pollen flow

External pollen flow, commonly referred to as pollen contamination, can affect the genetic composition of SO crops (Lai et al. 2010; Song et al. 2018). In our framework, pollen contamination is incorporated into the optimization by allowing external pollen donors to contribute to the SO crop exclusively through the male function (Funda and El-Kassaby 2012). In this setting, external donors contribute only through the male component, while orchard parents retain both female and male functions. This distinction can be handled in two ways within the optimization framework. One is an explicit formulation in which female and male gametic contributions are modeled separately through *f* and *m*. The other is an aggregate formulation in which pollen contamination is incorporated by modifying the “statusnumber” constraint. The aggregate formulation has the practical advantage of reducing model complexity and computational burden, while still capturing the overall effect of contamination on effective contribution and coancestry. In the present study, the optimization analyses were based on the aggregate formulation.

Under this formulation, maternal gametic contribution was assumed to originate entirely from orchard parents, whereas a proportion *C* of the paternal gametic contribution was assumed to originate from outside the SO. SO parents therefore account for the full maternal half of the SO crop and for only a proportion 1−C of the paternal half. Their total effective genetic contribution is thus


(7) $$\sum_{i=1}^{n_{c}}p_{i}=1-\frac{C}{2}.$$

To account for the effect of contamination on diversity, we followed the group coancestry formulation for SO crops with pollen contamination (Lindgren and Mullin 1998). In this formulation, the contribution of external pollen to group coancestry is represented as $M^{2}\theta_{MM}$, where *M* is the fraction of the seed crop gene pool originating from external pollen and $\theta_{MM}$ is the coancestry between external pollen gametes. Because the paternal gametic contribution represents half of the seed crop gene pool, a pollen contamination rate of *C* corresponds to a total gene pool fraction of M=C/2 from external pollen. Assuming $n_{d}$ unrelated external pollen donors with equal contribution gives $\theta_{MM}=1/(2n_{d})$, and therefore $M^{2}\theta_{MM}=(C/2{)}^{2}(1/(2n_{d}))=C^{2}/(8n_{d})$. Accordingly, the within-SO coancestry term was constrained as


(8) $$\sum_{i=1}^{n_{c}}\sum_{\;j=1}^{n_{c}}c_{ij}p_{i}p_{j}\leq \frac{1}{2n_{s}}-\frac{C^{2}}{8n_{d}}.$$

After optimization, the total expected genetic gain under pollen contamination was evaluated as


(9) $${\mathrm{Gain}}_{\mathrm{total}}=\sum_{i=1}^{n_{c}}p_{i}b_{i}+\frac{C}{2}{\bar{b}}_{\mathrm{ext}},$$

where ${\bar{b}}_{\mathrm{ext}}$ denotes the mean breeding value of the external pollen donors. Further implementation details for both formulations are provided in the Supplementary Material.

### Model input and execution workflow

As previously highlighted, one of AMPL’s strengths lies in its ability to separate the model and data into distinct files. In the referenced AMPL code (Box 2), the command on line 15 serves to initiate the data mode. The subsequent lines from 16 to 19 furnish an example of specific parameter values.

Box 2 **OptOrch.dat**

**Table:** 

data;	15
param ns := 30;	16
param cu := 0.5;	17
param nc := 500;	18
param nd := 20;	19

Based on our experience, we advocate for keeping all vector and matrix parameters in distinct data files, a topic that will be elaborated on in the subsequent section.

In the AMPL command script (Box 3), we undertake preparatory steps followed by the actual optimization process. In this workflow, AMPL is responsible for model declaration and problem instantiation, whereas Gurobi serves as the numerical solver that computes the optimal solution. The code initiates by directing AMPL to reset the computing environment (line 20), then proceeds to load the model specified in the ’OptOrch_core.mod’ file (line 21). Subsequently, we upload the scalar values, previously provided in Box 2 (line 22). Thereafter, we import three vectors, each stored in separate data files: ’b.dat’ contains a column of breeding values (line 23), while ’l.dat’ and ’u.dat’ provide columns of lower and upper bounds on *p*, respectively (lines 24-25). Next, we upload a coancestry matrix from the ’c.dat’ file (line 26), ensuring all data apply to the individuals within set S.

Box 3 **OptOrch.run**

**Table:** 

reset;	20
model OptOrch_core.mod;	21
data OptOrch.dat;	22
data b.dat;	23
data l.dat;	24
data u.dat;	25
data c.dat;	26
option solver GUROBI;	27
solve;	28
display gain;	29
display p;	30

With the model and data components prepared, a suitable solver is selected based on the problem’s specifics. We used Gurobi in this study because it provides strong computational performance and supports the mixed-integer quadratic features required by the OptOrch_core.mod formulation. However, the AMPL-based framework can also be linked to open-source solvers when they support the relevant problem class. For example, CBC (Forrest and Lougee-Heimer 2005) may be suitable for linear or mixed-integer linear variants, whereas SCIP (Achterberg 2009) may support broader quadratic or nonlinear formulations, depending on the formulation and interface. The ’solve’ command (line 28) directs AMPL to construct a specific optimization problem instance using the supplied model and data. After a review for potential errors, AMPL embarks on a presolve phase to simplify the model for the solver. The problem is then passed to the solver and, upon execution completion, the final values are displayed to the user (lines 29, 30). For a more comprehensive understanding of the AMPL syntax, refer to Fourer et al. (2003).

Up until now, our attention has been primarily on the optimization process itself, without explicit reference to a particular API. The crucial takeaway is that regardless of the interface used, the coding techniques we have introduced remain consistent. APIs offer the advantage of allowing models to be run from external programs, facilitating direct data and results exchange between AMPL’s model entities and the data structures of the external language. As of now, AMPL integrates with Python, R, C++, C#, MATLAB, and Java. However, in our examples, we have opted for simplicity by using the desktop console, which can be conveniently downloaded alongside the AMPL software.

Although the same optimization problem can also be assembled directly through the Gurobi interface in R, that route requires solver-specific declaration of all model components and is therefore substantially less concise than the AMPL formulation presented here. We include this distinction to clarify that OptOrch is centered on solver-independent model specification in AMPL, while R is used mainly for workflow automation and comparative benchmarking.

### Simulation scenarios

Having established the mathematical models and computational framework using AMPL, we now demonstrate the tool’s practical utility. To evaluate the framework’s performance under realistic operational conditions, we applied our optimization models to a simulated tree breeding dataset. The simulation analyses described in this section are provided in the public repository as R-based solver-specific scripts using Gurobi R interface and can be executed without running AMPL. In the following sections, we outline the generation of this simulated population and present optimization scenarios designed to test the algorithm’s computational efficiency, its response to varying genetic diversity constraints, and its robustness in the face of external biological factors.

#### Simulated population and genetic evaluation

A simulated diploid founder population consisting of 5,000 individuals was generated using the R package MoBPS (Pook et al. 2020). The genome comprised four chromosomes of equal length 100 cM, containing 20,300 SNPs. Among these, 300 were randomly assigned as quantitative trait loci (QTLs) controlling the simulated trait, while the remaining 20,000 loci were used as marker SNPs for genetic evaluation. QTL loci were excluded from the set of SNP markers to mimic realistic scenarios where causal variants are unknown. Allele frequencies for both SNPs and QTLs were sampled from a beta distribution Beta(α=1,β=1). Additive QTL effects were sampled from a normal distribution, $N(0,\sigma_{a}^{2})$, where $\sigma_{a}^{2}$ denotes the additive genetic variance. To establish a base population with realistic linkage disequilibrium patterns among markers, the founder population underwent 100 generations of random mating prior to selection. This procedure allowed recombination and genetic drift to generate non-random associations among loci.

For each individual, the true breeding value (TBV) was defined as the sum of additive effects across QTLs. The total additive genetic variance was standardized to one. Phenotypes were subsequently generated according to the linear model:


(10) $$y_{i}=\mu +TBV_{i}+e_{i},$$

where *μ* denotes the overall mean, and $e_{i}$ represents a normally distributed environmental residual term. The residual variance was determined according to the designated narrow-sense heritability ($h^{2}=0.2$). 100 individuals with the highest simulated phenotypic values were selected from the base population as parental trees and were randomly mated to generate 500 candidate individuals.

Genomic relationship matrices were computed from SNP genotypes using the method of VanRaden (VanRaden 2008), defined as:


(11) $$G=\frac{ZZ^{⊤}}{2\sum_{k=1}^{m}p_{k}(1-p_{k})},$$

where *Z* denotes the genotype matrix centered by allele frequencies, $p_{k}$ is the allele frequency at marker *k*, and *m* denotes the total number of marker loci.

Genetic evaluation was conducted using ASReml-R (Butler et al. 2023), which fits linear mixed models using the restricted maximum likelihood approach to estimate the variance components and predict the breeding values via the genomic best linear unbiased prediction (GBLUP). The following GBLUP model was fitted as y=μ+u+e, where *y* is the vector of phenotypes, *μ* the overall mean, *u* the vector of additive genetic effects, and *e* the residual term. The additive effects were assumed to follow $u∼N(0,G\sigma_{a}^{2})$, where G is the genomic relationship matrix constructed using the VanRaden method, and $\sigma_{a}^{2}$ denotes the additive genetic variance. The residuals were assumed to follow $e∼N(0,I\sigma_{e}^{2})$, where I is the identity matrix and $\sigma_{e}^{2}$ represents the residual variance. The predicted random effects $\hat{u}$ were regarded as GEBVs.

#### Optimization scenarios: genetic constraints and pollen contamination

In this section, we elucidate the fundamental operations of the proposed optimization framework employing the simulated population that closely mirrors the real-world scenarios observed in forest tree breeding. The objective is to demonstrate how the model responds to different breeding scenarios, especially genetic constraints and external pollen contamination, thereby providing an intuitive understanding of its practical applicability.

In the first scenario, the optimization focuses solely on genotype selection, genetic gain, and maintenance of genetic diversity. As shown in the flow chart (Fig. 1), the GEBVs and genetic coancestry matrix *c* from the simulated candidate population are subsequently used as inputs for the optimization. The genetic constraint is imposed as a limit on the $n_{s}$. Four levels of $n_{s}$ (10, 20, 30, and 40) are evaluated to examine how increasing diversity constraints influence the optimal contribution of candidate individuals. These values were chosen to illustrate a range of gain–diversity trade-offs, from relatively strong selection intensity at $\left(n_{s}=10\right)$ to stricter diversity maintenance at $\left(n_{s}=40\right)$. The appropriate value in practical applications is program-specific and depends on the deployment objective, relatedness among candidates, and acceptable balance between short-term gain and genetic diversity. The lower bound *l* and the upper bound *u* of individual contributions are set to 0.01 and 0.15, respectively, ensuring that each selected parent contributes at least 1% and at most 15% to the total number of clonal individuals established in the SO.

**Fig. 1.**
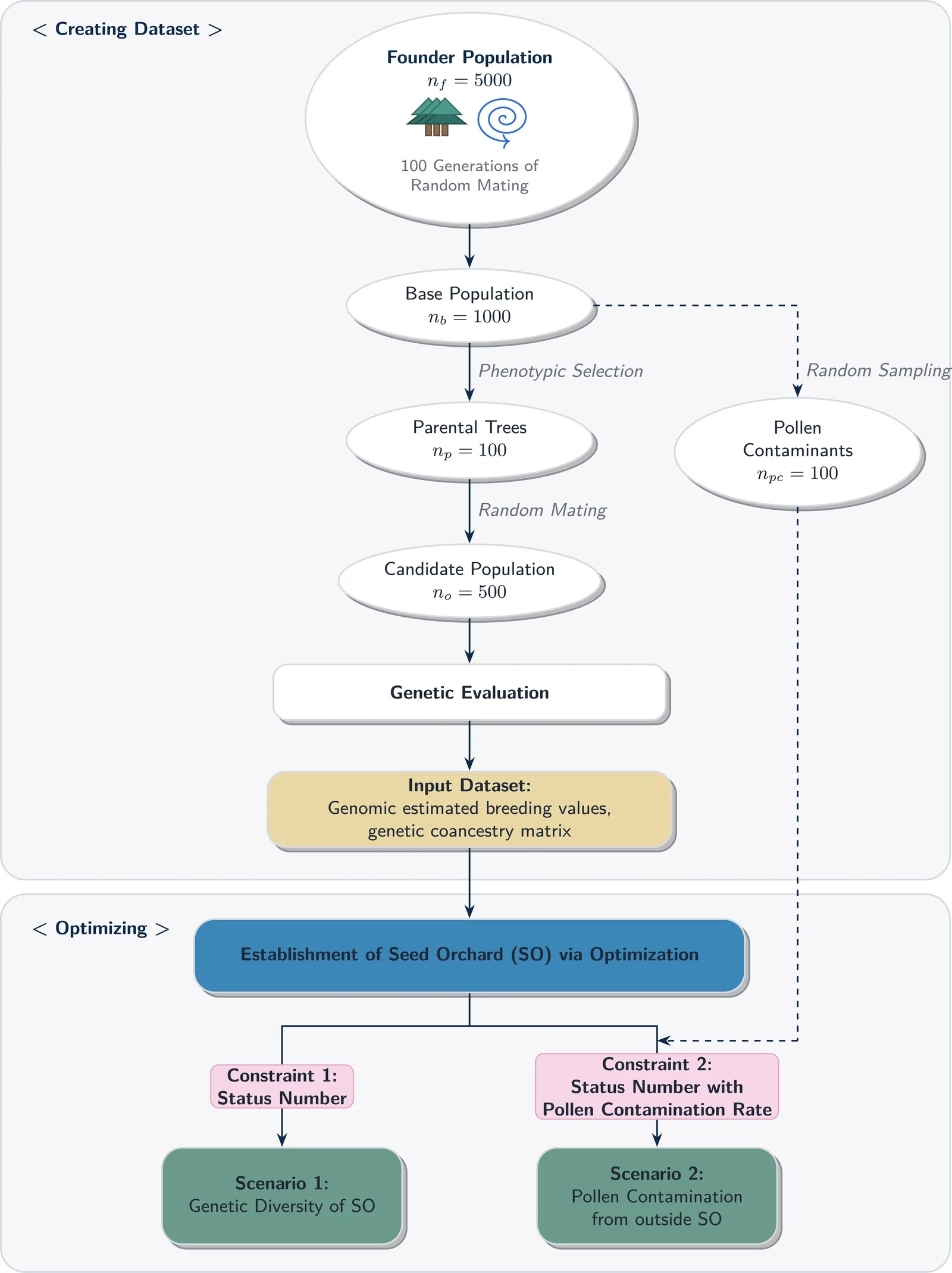
Overall workflow of the proposed optimization framework. Oval shapes represent populations. A founder population underwent 100 generations of random mating to establish linkage disequilibrium, resulting in a base population. From this base population, 100 parental trees were selected and randomly mated to generate a candidate population of 500 individuals for genetic evaluation. Pollen contamination was simulated by randomly sampling 100 individuals from the base population. Genomic estimated breeding values and the genetic coancestry matrix were used as inputs for optimization. SO establishment was evaluated under two scenarios.

In the second scenario, a pollen contamination rate of 30% from $n_{d}=100$ external trees was incorporated to mimic more realistic SO conditions. Accordingly, the external pollen contamination rate was set to C=0.3, corresponding to an effective contribution of C/2=0.15 to the seed-crop gene pool. Consequently, the total clonal contribution within the SO *p* was reduced to 0.85. By comparing this scenario with the previous case without pollen contamination, we assess how the external pollen influx influences the expected genetic response under identical $n_{s}$. Together, these two scenarios allow us to disentangle the effects of the internal genetic constraints and the external biological factors on optimal contribution strategies, providing insight into how SO design should be adjusted under operational conditions.

The optimization inputs consisted of the GEBVs and the matrix *c* of the candidate population. The genomic relationship matrix G, which was used for genetic evaluation and GEBV estimation, was scaled by 0.5 to obtain *c* for use in the status-number constraint. The optimization model was defined in AMPL, whereas R was used to generate the simulated data, prepare inputs, automate scenario analyses, and summarize outputs. In all analyses, Gurobi served as the numerical solver, accessed either through AMPL, which represents the primary OptOrch workflow, or through solver-specific input in R for comparison.

Because solver performance depends on the specific optimization instance, that is, the combination of model formulation and input data, we first examined solver tuning as a computational demonstration. The purpose was not to derive universally optimal parameter settings, but to illustrate how strongly tuning can affect run time and convergence for a given OptOrch problem. Parameter tuning was therefore performed with the Gurobi tuning tool under a 3,600 s time limit per configuration and a total tuning time of 86,400 s. All computations were performed on an Intel Core Ultra 7 265H CPU with 16 GB RAM under Windows 11 Home using R version 4.5.2 and Gurobi version 13.0.0. Optimization runs were executed sequentially, and the number of Gurobi threads was not manually specified. Under the baseline configuration, *MIPGap* was set to 0.005 and *NonConvex* to 2. *MIPGap* specifies the relative optimality gap tolerance used to terminate the optimization, whereas *NonConvex* enables Gurobi to solve models with nonconvex quadratic constraints. The tuned parameter set identified for each $n_{s}$ was then used in the subsequent scenario analyses.

After optimization, expected genetic response and diversity metrics were summarized for each scenario. Expected genetic response, representing the expected genetic gain from the optimized deployment, was defined as the contribution-weighted mean breeding value of the selected candidates under the optimized contribution vector. Under idealized open-pollination assumptions, this value corresponds to the expected mean breeding value of the resulting seed crop. In the no-contamination scenario, expected genetic response was calculated as the optimized objective value defined in Equation 1, $\sum_{i}p_{i}b_{i}$. In the pollen-contamination scenario, expected genetic response was calculated as the total expected genetic gain, as defined in Equation 9, $\sum_{i}p_{i}b_{i}+(C/2){\bar{b}}_{\mathrm{ext}}$, thereby accounting for the expected contribution of external pollen donors.

For diversity metrics, realized group coancestry in the no-contamination scenario was calculated as the left-hand side of Equation 2, evaluated using the optimized contribution vector, and is denoted as $\Theta_{\mathrm{orchard}}$. In the pollen-contamination scenario, realized group coancestry was evaluated at the total seed-crop level by including the expected coancestry contribution of the external pollen pool, following Lindgren and Mullin (1998):


(12) $$\Theta_{\mathrm{total}}=\Theta_{\mathrm{orchard}}+\frac{C^{2}}{8n_{d}}.$$

For each scenario, the realized status number was calculated following Lindgren et al. (1996):


(13) $$N_{s,\mathrm{realized}}=\frac{1}{2\Theta },$$

where *Θ* denotes $\Theta_{\mathrm{orchard}}$ for the no-contamination scenario and $\Theta_{\mathrm{total}}$ for the pollen-contamination scenario.

## Results

### Computational performance and solver tuning

Because all case-study optimizations used Gurobi as the numerical backend, we begin by reporting solver tuning results. These results are presented as a computational demonstration, showing how strongly solver behavior can depend on a particular OptOrch problem instance and how much performance can improve after tuning. Performance improvements varied across $n_{s}$, as shown in Table 1 with the configurations of the parameters and the run time for each $n_{s}$. The optimal tuning configuration reflected the structural changes in $n_{s}$. For all $n_{s}$, the adjustment substantially reduced the optimization time compared to the default solver settings.

**Table 1.** Optimization time and tuned Gurobi parameter settings for each status number ($n_{s}$).

		Description	$n_{s}=10$	$n_{s}=20$	$n_{s}=30$	$n_{s}=40$
Optimization time	Baseline time (s)	Optimization time using the baseline Gurobi settings	0.87±0.02	4.87±0.34	25.32, 24.51, $>{3,600}^{*}$	>3,600 , >3,600, >3,600
	Tuned time (s)	Optimization time using the tuned parameter configuration	0.26±0.00	0.50±0.00	0.75±0.02	929.11±5.04
Parameter	Aggregate	Controls the aggregation level in presolve	0	–	0	–
	NormAdjust	Chooses the simplex pricing norm	0	–	–	–
	NumericFocus	Controls the emphasis on numerical accuracy over speed	3	–	–	–
	PreMIQCPForm	Controls the format of the presolved MIQCP model	1	–	1	–
	ScaleFlag	Controls model scaling	2	0	0	2
	Heuristics	Determines the amount of time spent in MIP heuristics	–	0.5	–	0.001
	MIQCPMethod	Controls the method used to solve MIQCP models	–	0	–	–
	PrePasses	Limits the number of presolve passes	–	1	–	–
	Presolve	Controls the presolve level	–	1	1	–
	CutPasses	Limits the number of cutting-plane passes during root cut generation	–	–	10	–
	Cuts	Controls global cut aggressiveness	–	–	1	–
	ZeroHalfCuts	Controls zero-half cut generation	–	–	0	–
	NoRelHeurTime	Limits the time spent in the NoRel heuristic before solving the root relaxation	–	–	–	900

Each parameter configuration was evaluated three times. For settings where all runs converged, computation time is reported as means ± standard errors. Timeout cases are reported separately. The tuned parameter values were identified using the Gurobi tuning procedure and then applied to the corresponding $n_{s}$ level. Dashes indicate parameters that were not manually adjusted beyond the baseline Gurobi settings for that $n_{s}$ level.

^*^, time over 3,600 s indicates cases where the optimization did not converge within the time limit; The baseline Gurobi settings used in all optimization runs were MIPGap = 0.005, OutputFlag = 1, and NonConvex = 2. MIPGap specifies the relative optimality gap tolerance, OutputFlag controls whether solver output is printed, and NonConvex enables Gurobi to solve models with nonconvex quadratic constraints; Computations were performed on an Intel Core Ultra 7 265H CPU, with 16 GB RAM, running Microsoft Windows 11 Home version 10.0.26100. Analyses were conducted in R version 4.5.2 using Gurobi version 13.0.0. Optimization runs were executed sequentially, and the number of Gurobi threads was not manually specified.

For the smallest $n_{s}$ ($n_{s}=10$), both feasible solutions and bounds were obtained efficiently under the default setting, but the optimal configuration was able to reduce the time by approximately 70%. The performance gain was mainly attributed to improved presolve control and numerical stability. Disabling aggregation (Aggregate=0) prevents excessive presolve transformations that may degrade numerical accuracy, while setting NormAdjust=0 stabilized the simplex pricing scheme. In addition, PreMIQCPForm=1 enforced a structured reformulation of quadratic constraints. For $n_{s}=20$, the optimization became more sensitive to the balance between presolve reduction and heuristic search. PrePasses=1 allowed an additional presolve pass, leading to further model simplification. Simultaneously, Heuristics=0.5 allocated more computational effort to feasibility heuristics, enabling the solver to obtain higher-quality incumbent solutions at an early stage. This improved pruning efficiency and accelerated convergence. At $n_{s}=30$, the solver began to encounter convergence difficulties under the default configuration. Two runs converged within approximately 25 s (25.3 and 24.5 s), whereas one run exceeded the 3,600 s time limit. In contrast, the tuned configuration achieved consistent convergence (0.75±0.02 s).

The tuning strategy focused on strengthening the relaxation. Activating Cuts=1 introduced additional cutting planes that tighten the linear relaxation, thereby improving the dual bound. Together with PreMIQCPForm=1, this improved the quality of the reformulation and facilitated faster bound convergence. For the largest scale ($n_{s}=40$), all default runs failed to converge within the 3,600 s limit. Although feasible solutions were likely identified, the solver struggled to close the optimality gap due to slow dual bound improvement. The tuned configuration shifted the search strategy toward improving the dual bound rather than investing effort in feasibility heuristics. Setting Heuristics=0.001 significantly reduced the time spent on heuristic solution search. In addition, ScaleFlag=2 applied more aggressive scaling to improve numerical conditioning, while NoRelHeurTime=900 enabled an initial feasibility search before solving the root relaxation. After obtaining a feasible solution, the solver focused on tightening the dual bound, which was the main difficulty at this scale. The optimal parameters differed across $n_{s}$, indicating structural changes in the search space as the constraint became more restrictive. Accordingly, the parameter set for each $n_{s}$ was used in all subsequent analyses.

### Optimization examples

Here we present the results of the two optimization scenarios used to demonstrate the functionality of the OptOrch software under representative deployment conditions. Because the lower bound *l* was set to 0.01, all optimal contributions remained at or above this threshold (Fig. 2). This constraint prevents trivial contributions that may arise from the solver to assign extremely small decimal values. In practical SO, clonal contributions cannot be represented as fractional individuals. In addition, the upper bound *u* prevents optimal contributions from being disproportionately allocated to a limited number of clones.

**Fig. 2.**
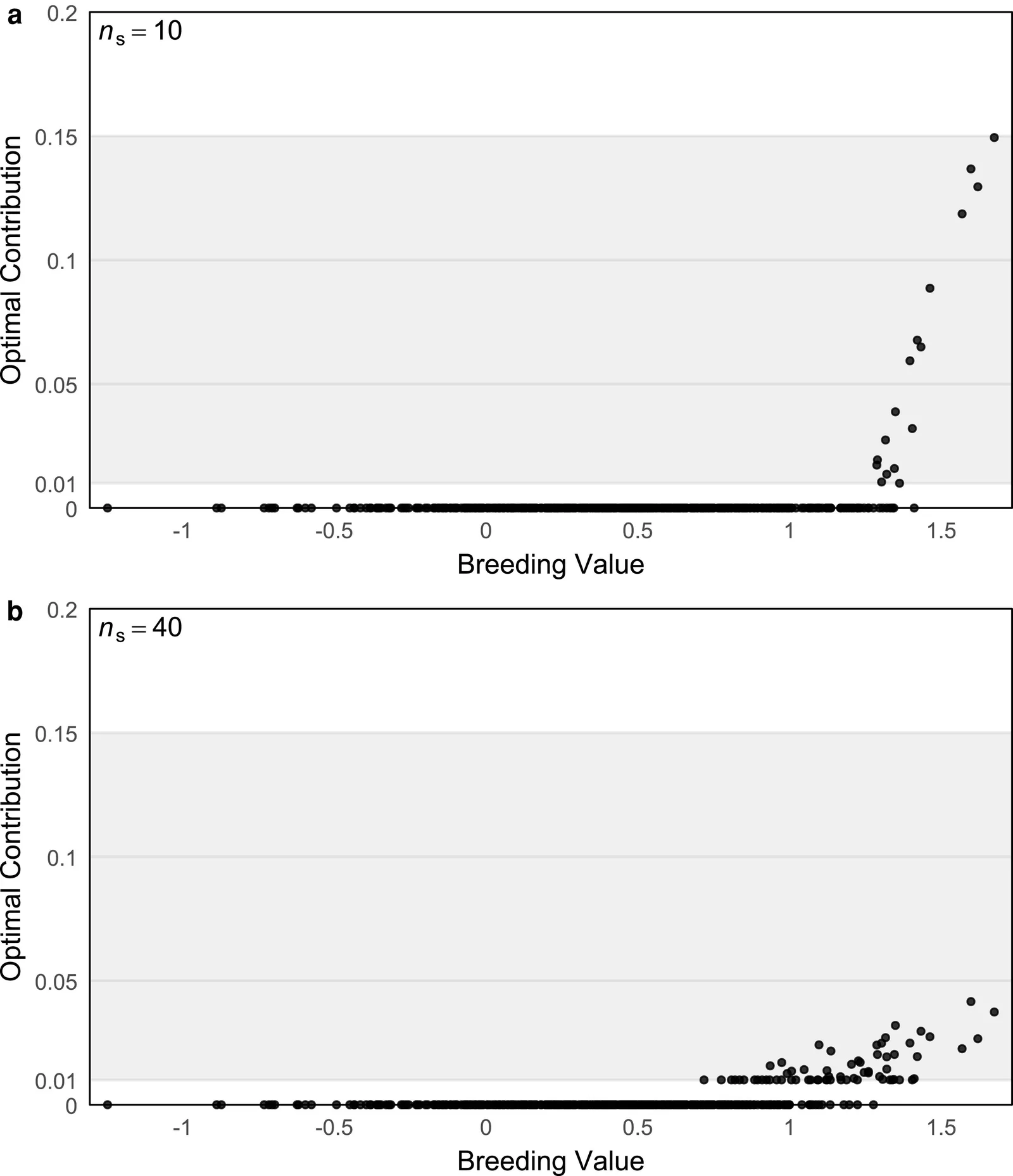
Optimization results for the contributions of the candidate individuals under different status numbers ($n_{s}$). Panel (a) shows the result for $n_{s}=10$, and panel (b) shows the result for $n_{s}=40$. The gray shaded region indicates the limited contribution range imposed by the scenario condition.

Optimal contributions changed markedly as the $n_{s}$ constraint increased from 10 to 40 (Fig. 2). Under $n_{s}=10$, contributions were highly concentrated among a relatively small number of top-ranking individuals. In the no-contamination scenario, the mean number of selected candidates was 19±1 at $n_{s}=10$, whereas it increased to 64±1 at $n_{s}=40$. Similarly, in the pollen contamination scenario, the corresponding number increased from 14±0 to 48±1 (Supplementary Table S1). Although individuals with higher breeding values still tended to receive larger contributions, the allocation became less extreme as $n_{s}$ increased.

Consistent with the changes observed in optimal contributions (Fig. 2), expected genetic response under the non-contamination scenario decreased as the $n_{s}$ increased (Fig. 3). When $n_{s}=10$, the expected genetic response was highest, reflecting the strong concentration of contributions among top-ranking individuals. As the $n_{s}$ constraint increased, contributions were spread across more candidates, reducing selection intensity and consequently lowering the short-term genetic gain.

**Fig. 3.**
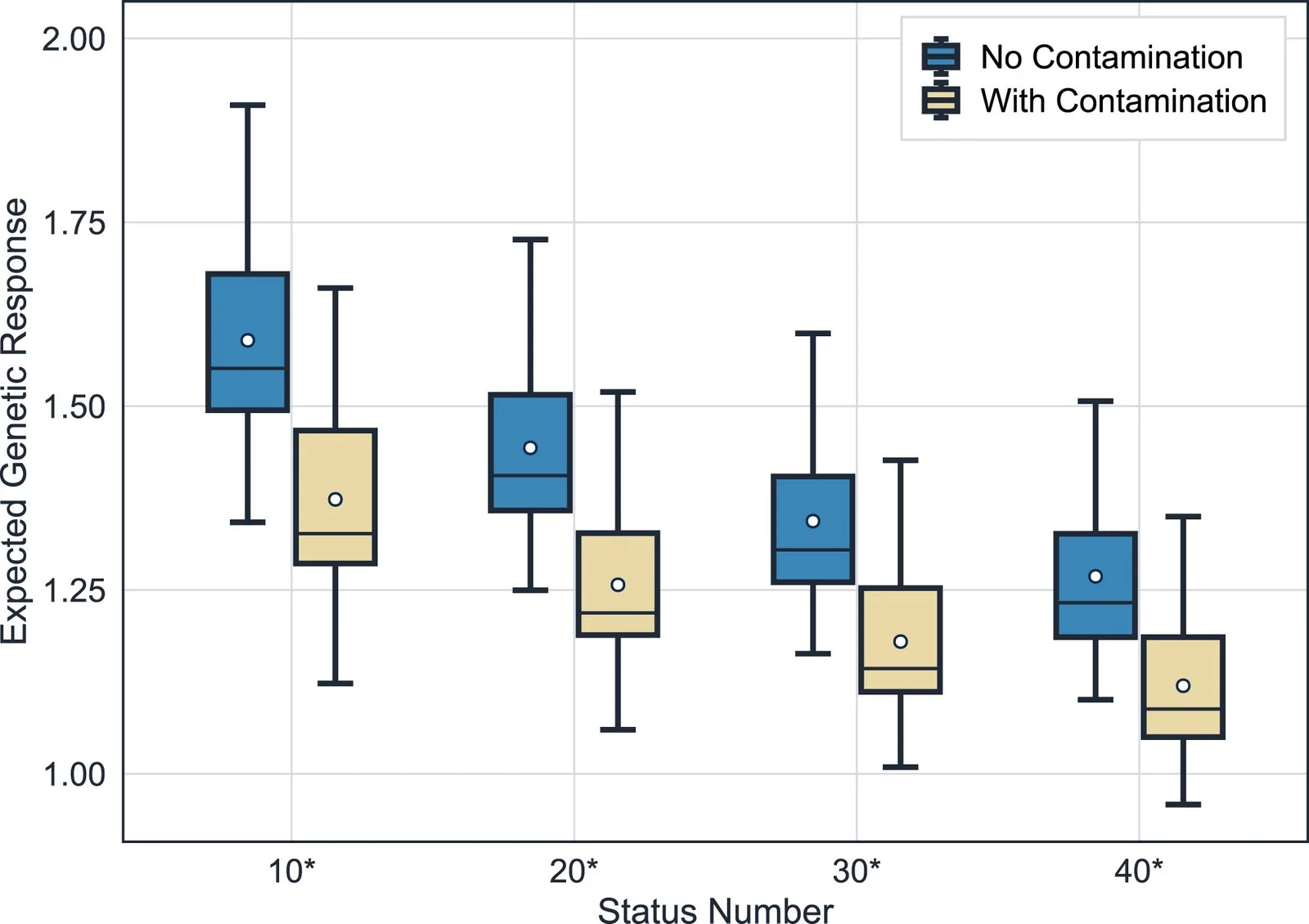
Effect of status number and pollen contamination on expected genetic response. Asterisks (*) denote statistically significant differences between the ’no contamination’ and ’with contamination’ scenarios according to the Mann–Whitney U test (p<.0001).

When pollen contamination was incorporated into the simulation, the expected genetic response under contamination remained at approximately 86–88% of that observed in the non-contamination scenario across all $n_{s}$ levels (Fig. 3). Since the total contribution within SO was limited to 0.85, the constraint on $n_{s}$ could be satisfied with a smaller number of selected clones and individuals, thereby increasing the selection intensity within the SO. Nevertheless, this adjustment was insufficient to compensate for the loss of genetic gain caused by external pollen, as a fixed proportion of the genetic contribution originated from unselected sources with lower genetic merit.

## Discussion

OptOrch provides a flexible and transparent framework for optimizing SO deployment under realistic biological and operational constraints. A central strength of the software is that the formulation can be fine-tuned by the user through simple algebraic modification of the model structure. Because the optimization problem is expressed in AMPL, the objective function, variables, and constraints remain explicit and closely aligned with standard mathematical notation (Fourer et al. 2003), allowing users without advanced expertise in mathematical programing to inspect, understand, and adapt the formulation to their own breeding objectives.

The use of mathematical programing also provides an important advantage over heuristic approaches. Solutions can be evaluated relative to the global optimum within a predefined optimality tolerance, offering a quantitative reference for solution quality that is generally unavailable in heuristic search (Gurobi Optimization, LLC 2026). When the declared formulation is supported by the selected solver and the solver terminates with an optimality certificate, the solution can be interpreted relative to the reported bound and optimality gap. This property is particularly relevant in SO deployment, where multiple biological and operational constraints interact and where the consequences of alternative decisions can be substantial (Prescher et al. 2008; Funda et al. 2009). In this sense, OptOrch serves not only as a deployment tool, but also as a decision-support framework for comparing competing strategies on a rigorous quantitative basis.

An important contribution of OptOrch lies not only in the optimization model itself, but also in the way it is implemented and exposed to the user. By separating the mathematical formulation, input data, and numerical solver, the software remains transparent, reproducible, and easier to modify than a direct solver-specific implementation (Fourer et al. 2003). In practical terms, users can revise deployment constraints or extend the model without having to reconstruct solver-native data structures. This separation is especially valuable in breeding applications, where objectives and constraints may change as new biological information or management priorities emerge.

The illustrative scenarios presented here demonstrate how the framework can be used to quantify trade-offs between genetic gain and diversity and to evaluate biologically relevant factors such as external pollen flow. The latter is of particular practical importance, because gene flow from external pollen donors may alter both the genetic composition and the realized gain of SO crops (El-Kassaby et al. 1989; Kang et al. 2001; Funda and El-Kassaby 2012). Accounting for such effects allows a more realistic assessment of expected orchard performance and avoids overly optimistic predictions based solely on within-orchard parental contributions.

The definition of the candidate set is an important practical decision. OptOrch optimizes over the candidates provided by the user; therefore, the input set can represent either the full available breeding-value dataset or a pre-filtered operational subset. When computationally feasible, providing the full candidate set allows the optimization to determine which individuals should receive non-zero contributions under the imposed gain, diversity, and operational constraints. In other situations, breeders may first exclude candidates that are unsuitable for deployment because of limited graft availability, poor propagation, disease, flowering constraints, or other biological management considerations. The simulated example used here treated the 500 offspring genotypes as the candidate set for optimization, representing a deployment decision in which selection and proportional contribution are determined jointly.

The present examples focus on open-pollinated clonal SO deployment, where the main decision is the composition of the orchard and the proportional representation of each clone. Controlled-pollination or full-sib seed production systems represent a related but distinct optimization problem. When male pollen is controlled, breeders may also need to optimize pollen allocation, female-by-male mating pairs, family representation, crossing capacity, and constraints on full-sib contributions. Under such systems, a smaller number of elite selections may be sufficient if diversity and inbreeding are managed explicitly through the crossing design, but this depends on the reproductive system, deployment objective, and operational constraints. The algebraic structure of OptOrch could accommodate such extensions by introducing sex-specific gametic contributions or mate-pair decision variables, but these formulations are outside the scope of the open-pollinated SO examples presented here.

The practical contribution of OptOrch is not the introduction of a new optimal contribution criterion, but the translation of existing gain-diversity deployment concepts into a modifiable algebraic modeling framework for SO management. Earlier forestry applications demonstrated the value of mathematical programing for selected orchard scenarios, and general optimal contribution tools remain useful for breeding applications that match their implemented structure. OptOrch addresses a complementary need by allowing SO-specific deployment assumptions to be modified directly in the model formulation, while leaving numerical optimization to an appropriate solver.

Finally, users should pay close attention to the input parameters and the combinations of imposed constraints. Infeasibility often arises from overly restrictive bounds or incompatible biological requirements. Exploring multiple scenarios can help clarify the feasible region and reveal the trade-offs implicit in a given deployment strategy. The value of OptOrch therefore lies not only in identifying high-performing solutions, but also in making the biological and operational consequences of those solutions explicit and quantitatively comparable.

## Supplementary Material

jkag208_Supplementary_Data

## Data Availability

All code and data required to reproduce the analyses presented in this manuscript are archived in the OptOrch version 1.0.1 release on Zenodo (Kim et al. 2026). The development repository is available at https://github.com/kyeji1107/OptOrch. The archive includes the AMPL model files, a runnable AMPL example, R scripts for MoBPS-based population generation and optimization, generated input datasets, optimization outputs, figure-generation scripts, and a locally run Shiny application. The software is distributed under the MIT License. Supplementary File S1 provides an overview of the repository, extended AMPL formulations, and Supplementary Table S1. Supplemental material available at G3 online.

## References

[jkag208-B1] Achterberg T . 2009. SCIP: solving constraint integer programs. Math Program Comput. 1:1–41. 10.1007/s12532-008-0001-1.

[jkag208-B2] Ahlinder J, Mullin T, Yamashita M. 2014. Using semidefinite programming to optimize unequal deployment of genotypes to a clonal seed orchard. Tree Genet Genomes. 10:27–34. 10.1007/s11295-013-0659-z.

[jkag208-B3] Butler D, Cullis B, Gilmour A, Gogel B, Thompson R. 2023. ASReml-R reference manual Version 4.2.

[jkag208-B4] El-Kassaby YA, Rudin D, Yazdani R. 1989. Levels of outcrossing and contamination in two *Pinus sylvestris* L seed orchards in Northern Sweden. Scand J For Res. 4:41–49. 10.1080/02827588909382544.

[jkag208-B5] Fernández J, Toro M. 1999. The use of mathematical programming to control inbreeding in selection schemes. J Anim Breed Genet. 116:447–466. 10.1046/j.1439-0388.1999.00196.x.

[jkag208-B6] Forrest J, Lougee-Heimer R. 2005. CBC user guide. INFORMS TutORials in operations research. p. 257–277.

[jkag208-B7] Fourer R, Gay D. 2002. Extending an algebraic modeling language to support constraint programming. INFORMS J Comput. 14:322–344. 10.1287/ijoc.14.4.322.2825.

[jkag208-B8] Fourer R, Gay D, Kernighan B. 2003. AMPL: a modeling language for mathematical programming. 2nd ed. Thomson Brooks/Cole. p. 517 + xxi.

[jkag208-B9] Funda T, El-Kassaby YA. 2012. Seed orchard genetics. CABI Rev. 7:1–23. 10.1079/PAVSNNR20127013.

[jkag208-B10] Funda T, Lstibůrek M, Lachout P, Klápště J, El-Kassaby YA. 2009. Optimization of combined genetic gain and diversity for collection and deployment of seed orchard crops. Tree Genet Genomes. 5:583–593. 10.1007/s11295-009-0211-3.

[jkag208-B11] Gorjanc G, Hickey JM. 2018. AlphaMate: a program for optimizing selection, maintenance of diversity and mate allocation in breeding programs. Bioinformatics. 34:3408–3411. 10.1093/bioinformatics/bty375. PMC6157072 29722792

[jkag208-B12] Gurobi Optimization, LLC . 2026. Gurobi optimizer reference manual.

[jkag208-B13] Hickey JM, Veerkamp RF, Calus MPL, Mulder HA, Thompson R. 2009. Estimation of prediction error variances via Monte Carlo sampling methods using different formulations of the prediction error variance. Genet Sel Evol. 41:23. 10.1186/1297-9686-41-23. PMC3225835 19284698

[jkag208-B14] Kang KS, Lindgren D, Mullin T. 2001. Prediction of genetic gain and gene diversity in seed orchard crops under alternative management strategies. Theor Appl Genet. 103:1099–1107. 10.1007/s001220100700.

[jkag208-B15] Kim YJ, Bittner V, Kang KS, Sagariya C, Lstibůrek M. 2026. OptOrch: a modular optimization toolkit for forest tree seed orchard deployment. Version 1.0.1. 10.5281/zenodo.21610348. PMC13643773 42538858

[jkag208-B16] Kinghorn BP . 1998. Mate selection by groups. J Dairy Sci. 81:55–63. 10.3168/jds.s0022-0302(98)70154-7. 9777512

[jkag208-B17] Lai BSK et al 2010. Pollination dynamics in a Douglas-fir seed orchard as revealed by pedigree reconstruction. Ann For Sci. 67:808. 10.1051/forest/2010044.

[jkag208-B18] Lindgren D, Gea L, Jefferson P. 1996. Loss of genetic diversity monitored by status number. Silvae Genet. 45:52–59.

[jkag208-B19] Lindgren D, Libby W, Bondesson F. 1989. Deployment to plantations of numbers and proportions of clones with special emphasis on maximizing gain at a constant diversity. Theor Appl Genet. 77:825–831. 10.1007/BF00268334. 24232899

[jkag208-B20] Lindgren D, Matheson AC. 1986. An algorithm for increasing the genetic quality of seed from seed orchards by using the better clones in higher proportions. Silvae Genet. 35:173–177.

[jkag208-B21] Lindgren D, Mullin TJ. 1998. Relatedness and status number in seed orchard crops. Can J For Res. 28:276–283. 10.1139/x97-217.

[jkag208-B22] Lindgren D, Wei R, Bondesson L. 1993. Optimal selection from families. Heredity (Edinb). 70:619–621. 10.1038/hdy.1993.88.

[jkag208-B23] Lstibůrek M, Hodge G, Lachout P. 2015. Uncovering genetic information from commercial forest plantations—making up for lost time using “breeding without breeding”. Tree Genet Genomes. 11:55. 10.1007/s11295-015-0881-y.

[jkag208-B24] Meuwissen T . 1997. Maximizing the response of selection with a predefined rate of inbreeding. J Anim Sci. 75:934–940. 10.2527/1997.754934x. 9110204

[jkag208-B25] Pong-Wong R, Woolliams JA. 2007. Optimisation of contribution of candidate parents to maximise genetic gain and restricting inbreeding using semidefinite programming. Genet Sel Evol. 39:3. 10.1186/1297-9686-39-1-3. PMC2739432 17212945

[jkag208-B26] Pook T, Schlather M, Simianer H. 2020. MoBPS-modular breeding program simulator. G3 (Bethesda). 10:1915–1918. 10.1534/g3.120.401193. PMC7263682 32229505

[jkag208-B27] Prescher F, Lindgren D, Karlsson B. 2008. Genetic thinning of clonal seed orchards using linear deployment may improve both gain and diversity. For Ecol Manage. 254:188–192. 10.1016/j.foreco.2007.08.014.

[jkag208-B28] R Core Team . 2025. *R: A Language and Environment for Statistical Computing*. R Foundation for Statistical Computing. Vienna, Austria.

[jkag208-B29] Resende MFRJ et al 2012. Accuracy of genomic selection methods in a standard data set of loblolly pine (*Pinus taeda* L.). Genetics. 190:1503–1510. 10.1534/genetics.111.137026. PMC3316659 22271763

[jkag208-B30] Song J et al 2018. Temporal quantification of mating system parameters in a coastal Douglas-fir seed orchard under manipulated pollination environment. Sci Rep. 8:11593. 10.1038/s41598-018-30041-4. PMC6072752 30072772

[jkag208-B31] VanRaden P . 2008. Efficient methods to compute genomic predictions. J Dairy Sci. 91:4414–4423. 10.3168/jds.2007-0980. 18946147

[jkag208-B32] Wellmann R . 2019. Optimum contribution selection for animal breeding and conservation: the R package optiSel. BMC Bioinformatics. 20:25. 10.1186/s12859-018-2450-5. PMC6332575 30642239

[jkag208-B33] Woolliams J, Berg P, Dagnachew B, Meuwissen T. 2015. Genetic contributions and their optimization. J Anim Breed Genet. 132:89–99. 10.1111/jbg.12148. 25823835

[jkag208-B34] Wu HX, Owen JV, Abarquez A, Matheson AC. 2004. Inbreeding in *Pinus radiata* - V. the effects of inbreeding on fecundity. Silvae Genet. 53:80–87. 10.1515/sg-2004-0015.

