# Supplementary material for: OptOrch: a modular optimization toolkit for forest tree seed orchard deployment
Source: G3 (Bethesda). 2026 Aug 1;16(10):jkag208. doi: 10.1093/g3journal/jkag208 (PMC13643773; doi:10.1093/g3journal/jkag208)
Supplement: Supplementary file 1 [file jkag208_supplementary_data.pdf]

Supplementary Material for OptOrch

Contents

1 Overview of repository files and reproducibility materials **2**

1.1 AMPL model files . . . 2

1.2 Minimal AMPL example . . . 2

1.3 R-based manuscript reproduction materials . . . 2

1.4 Local-run Shiny app . . . 3

1.5 Execution options . . . 3

2 Extended AMPL formulation variants **4**

2.1 Logical-operator formulation for selection-dependent bounds . . . 4

2.2 Constraining pairwise coancestry . . . 4

2.3 Female and male contributions . . . 5

2.4 Pollen contamination . . . 5

2.4.1 Explicit female–male formulation . . . 5

2.4.2 Aggregate formulation . . . 6

3 Supplementary Table **7**

1 Overview of repository files and reproducibility materials

The OptOrch GitHub repository provides the supplementary code, example files, and reproducibility materials for the optimization framework described in the manuscript. The repository is organized into four main components: AMPL model files, a minimal AMPL example, R-based manuscript reproduction materials, and a local-run Shiny app.

1.1 AMPL model files

The AMPL model files are provided in `01_models/`. This directory contains the core optimization model and six tailored model variants corresponding to the formulation extensions discussed in the manuscript.

- `01_models/001_core/OptOrch_core.mod`: core AMPL formulation for maximizing genetic gain under the status-number constraint and practical contribution bounds.
- `01_models/002_tailored/OptOrch_supp1.mod`: logical-operator formulation for selection-dependent bounds.
- `01_models/002_tailored/OptOrch_supp2.mod`: logical formulation for constraining pairwise coancestry.
- `01_models/002_tailored/OptOrch_supp3.mod`: linearized formulation for constraining pairwise coancestry.
- `01_models/002_tailored/OptOrch_supp4.mod`: formulation with separate female and male contributions.
- `01_models/002_tailored/OptOrch_supp5.mod`: explicit female–male formulation for pollen contamination.
- `01_models/002_tailored/OptOrch_supp6.mod`: aggregate formulation for pollen contamination.

1.2 Minimal AMPL example

A minimal AMPL example is provided in `02_examples/001_minimal_AMPL_example/`. This example demonstrates the basic AMPL workflow using separated input files, an integrated data file, and a run script.

- `OptOrch_core.mod`: core AMPL model file used in the example.
- `OptOrch.dat`: integrated data file specifying the main scalar parameters.
- `OptOrch.run`: AMPL run script for executing the example.
- `b.dat`: input file containing breeding values.
- `l.dat`: input file containing lower contribution bounds.
- `u.dat`: input file containing upper contribution bounds.
- `c.dat`: input file containing the coancestry matrix.

1.3 R-based manuscript reproduction materials

The R-based reproducibility materials are provided in `03_manuscript_reproduction/`. These files allow users to reproduce the simulation-based analyses and figures presented in the manuscript without relying on AMPL.

- `001_simulated_data/`: simulation code and MoBPS-generated input data.
- `002_scenario1_status_number/`: R script and outputs for the status-number scenario.
- `003_scenario2_pollen_contamination/`: R script and outputs for the pollen-contamination scenario.
- `004_figures/`: scripts and output files for reproducing Figures 2 and 3.

1.4 Local-run Shiny app

A local-run Shiny app is provided in `04_shiny_app/`. The app provides a graphical user interface for running the two manuscript optimization scenarios using the user's local R environment and local Gurobi installation.

- `app.R`: main Shiny app file.
- `README.md`: guide for launching and using the app.
- `Input_data/`: example input folder for user-provided breeding values and coancestry matrices.

1.5 Execution options

The repository supports three execution routes. First, users can run the AMPL-based example using the supplied model, data, and run files in `02_examples/001_minimal_AMPL_example/`. Second, users can reproduce the manuscript scenarios directly in R using the materials in `03_manuscript_reproduction/`. Third, users can run the two optimization scenarios through the local Shiny interface in `04_shiny_app/`.

2 Extended AMPL formulation variants

2.1 Logical-operator formulation for selection-dependent bounds

Some solvers support logical operators that allow the selection-dependent bounds to be written more compactly, thereby eliminating the auxiliary binary variable r from the core formulation. In the core model, this formulation replaces the declaration of the auxiliary variable r (line 9) together with the `lowerbound` and `upperbound` constraints (lines 13–14). In AMPL, the simplified constraint can be written directly using a logical operator, as shown below.

Box 1: `OptOrch_supp1.mod`

```
#Constraints
s.t. selectiondependentbounds {i in S}:
1      p[i] == 0 or l[i] <= p[i] <= u[i];
```

2.2 Constraining pairwise coancestry

Although the group-coancestry constraint in the core model controls the overall diversity of the selected set, breeders are often additionally concerned about pairwise coancestry between individual clones. To address this, an upper limit c_u can be imposed on acceptable pairwise coancestry among selected clones.

One intuitive implementation uses a logical operator, as shown below.

Box 2: `OptOrch_supp2.mod`

```
#Parameters and sets
2 param cu >= 0, <= 1;

#Constraints
s.t. pairwisecoancestry {i in S, j in S: i < j}:
3      if (p[i] * p[j]) > 0 then c[i,j] <= cu;
```

In this formulation, the pairwise coancestry $c_{i,j}$ between clones i and j must not exceed the threshold c_u when both clones receive non-zero contributions. However, this creates a non-convex constraint. Although modern solvers have made substantial progress in handling non-convex problems (Lundell and Kronqvist, 2022; Elloumi and Lambert, 2019), we also considered an alternative linearized implementation.

Box 3: `OptOrch_supp3.mod`

```
#Variables
4 var y {i in S, j in S} binary;

#Constraints
s.t. pairwiseexcludej {i in S, j in S : i < j and c[i,j] > cu}:
5      p[j] <= 1 - y[i,j];
s.t. pairwiseexcludei {i in S, j in S : i < j and c[i,j] > cu}:
6      p[i] <= y[i,j];
```

In this version, the auxiliary binary variable y (line 4) ensures that at most one clone is selected from each pair whose pairwise coancestry exceeds c_u. The constraints in lines 5–6 therefore allow the selection of clone i, clone j, or neither, but not both simultaneously.

If the traditional identity-by-descent (IBD) matrix is used, setting $c_u = 0$ completely excludes pairwise coancestry among selected clones. When a genomic relationship matrix is used, c_u may instead be set to a small positive value to allow limited relatedness. More generally, c_u may also be specified as a matrix-valued parameter if different upper bounds are required for different clone pairs.

2.3 Female and male contributions

To accommodate practical scenarios in which female and male gametic contributions must be treated separately, clonal contribution p was decomposed into female $f \in [0, 1]$ and male $m \in [0, 1]$ components. This formulation is useful for representing situations such as unequal female and male reproduction and pollen contamination. To ensure biological interpretability, these components are normalized so that the female contributions sum to 1 across orchard parents, whereas male contributions can later be partitioned between orchard parents and external pollen donors according to the specified contamination rate. As a result, clonal contributions can be expressed as constraint `femaleandmalecontribution`.

Box 4: `OptOrch_supp4.mod`

```
#Variables
var f {i in S} >= 0, <= 1;
var m {i in S} >= 0, <= 1;

#Constraints
s.t. femaleandmalecontribution {i in S}:
     p[i] = (f[i]+m[i])/2;
```

2.4 Pollen contamination

Pollen contamination was implemented by introducing an additional set of external pollen donors. To maintain model flexibility, users can specify the number of such external pollen donors, together with their corresponding coancestry and breeding values. Accordingly, we assume n_d trees that contribute exclusively as male parents. These trees are incorporated into the previously defined set S, which first comprises the trees present in the SO, followed by the external pollen donors. Their relatedness and breeding values are specified in the corresponding entries of the parameters c and b.

Pollen contamination can be represented in two related ways within the optimization framework. The first is an explicit formulation, in which female and male gametic contributions are modeled separately for each parent. The second is an aggregate formulation, in which the effect of contamination is incorporated indirectly through adjusted contribution and coancestry constraints.

2.4.1 Explicit female–male formulation

In the explicit formulation, the original set S is augmented to include the external pollen donors. Accordingly, n_c denotes the total number of individuals in the augmented set. The first $n_c - n_d$ indices represent the resident SO candidate parents, whereas the final n_d indices represent the external pollen donors. This indexing convention allows contributions, breeding values, and coancestry values for both groups to be handled within a single set.

Box 5: `OptOrch_supp5.mod`

```
#Parameters and sets
10 param nd > 0;

#Constraints
s.t. nofemaleexternal {i in (nc-nd+1)..nc}:
11      f[i] = 0;
s.t. orchardparent {i in 1..(nc-nd)}:
12      p[i] = (f[i]+m[i])/2;
s.t. externaldonor {i in (nc-nd+1)..nc}:
13      p[i] = m[i]/2;
```

Female contributions (f) pertain only to SO parents (line 11). For orchard parents, the total genetic contribution to the seed crop is defined as the average of the female and male gametic contributions (line 12). External pollen donors contribute only through the male function. Because the paternal gametic pool represents one-half of the seed-crop gene pool, the effective total contribution of an external donor is $m_i/2$ (line 13). To represent a specified pollen contamination rate C, the male contributions must be specified or constrained so that external donors jointly contribute C to the paternal gametic pool. Their joint effective contribution to the seed-crop gene pool is therefore $C/2$, while orchard parents jointly contribute $1 - C/2$. The resulting contributions p are propagated through the objective function and the coancestry constraint. The

aggregate formulation presented below incorporates the same relationship without modeling female and male contributions separately.

2.4.2 Aggregate formulation

In the aggregate formulation, external pollen donors are not represented individually in S; therefore, S and n_c retain their original definitions for the resident SO candidate population. Under this formulation, pollen contamination was assumed to act only through the paternal side, and the contaminating pollen pool was assumed to consist of mutually unrelated external donors contributing equally.

Box 6: `OptOrch_supp6.mod`

```
#Parameters
14 param nd > 0;
15 param C > 0, < 1;

#Constraints
s.t. effectivecontribution:
16      sum {i in S} p[i] = 1 - C/2;
s.t. contamstatusnumber:
        sum {i in S, j in S} c[i,j] * p[i] * p[j]
17      <= (1 / (2 * ns)) - (C^2 / (8 * nd));
```

In this implementation, external pollen donors are not introduced as decision variables. Instead, their effect is incorporated through the reduced effective contribution of orchard parents and the adjusted coancestry constraint. Lines 16 and 17 replace the core total-contribution and status-number constraints, respectively, and must not be imposed simultaneously with the original constraints.

3 Supplementary Table

Table S1. Effects of status number (n_s) and pollen contamination on expected genetic response and genetic diversity. Values are reported as means ± standard errors across 30 replicates for each n_s.

Scenario		Number of Selected Individuals	Expected Genetic Response	Group Coancestry	Realized Status Number
Contamination	n_s				
No Contamination	10	19 ± 1	1.589 ± 0.029	0.04980 ± 0.00007	10.04 ± 0.01
	20	35 ± 1	1.443 ± 0.025	0.02500 ± 0.00000	20.00 ± 0.00
	30	48 ± 1	1.344 ± 0.023	0.01667 ± 0.00000	30.00 ± 0.00
	40	64 ± 1	1.269 ± 0.021	0.01250 ± 0.00000	40.00 ± 0.00
With Contamination	10	14 ± 0	1.373 ± 0.026	0.04957 ± 0.00011	10.09 ± 0.02
	20	23 ± 1	1.257 ± 0.023	0.02500 ± 0.00000	20.00 ± 0.00
	30	36 ± 1	1.180 ± 0.021	0.01667 ± 0.00000	30.00 ± 0.00
	40	48 ± 1	1.120 ± 0.020	0.01250 ± 0.00000	40.00 ± 0.00

References

Elloumi S, Lambert A. 2019. Global solution of non-convex quadratically constrained quadratic programs. Optimization methods and software. 34:98–114.

Lundell A, Kronqvist J. 2022. Polyhedral approximation strategies for nonconvex mixed-integer nonlinear programming in shot. Journal of Global Optimization. 82:863–896.
